# Psoas Abscess

**Published:** 1895-01

**Authors:** Chas. A. Jefferies

**Affiliations:** Home, S. C.


### *CORRESPONDENCE.*

---

### PSOAS ABSCESS.

HOME, S. C., December 10, 1894.

*To the Atlanta Medical and Surgical Journal:*

On September 20, being summoned to see Ben. G., age twenty, weight one hundred and sixty pounds, a stout, robust negro, I found his temperature $100\frac{1}{5}°$, pulse 84, respiration 20, face anxious, and his right leg contracted on abdomen.

In the right inguinal region there was slight swelling, the muscles were somewhat hard and contracted, though palpation revealed sounds normal over the cecum and colon.

Hot fomentations, with flaxseed poultices, were applied every two hours, and morphine and antikamnia were given to allay the pain every three to four hours. No purgatives were allowed, enemata being used instead, but no improvement; pain continuing, swelling more marked, though choral and double amount of hot applications were used.

An incision, under cocaine, was made on the fifth day, one inch long, one and half deep, parallel to Poupart's ligament, two and one-fourth inches above, though there was no deep-seated tenderness to indicate pus; yet this seemed to be near the thickest portion of the swelling.

No pus was found, but an ounce and a half of grumous, clotted blood was removed, and the wound, after washing with hot water, was packed with iodoform gauze.

The swelling and tenderness had completely subsided on the fourth day; the wound was dressed and no trace of any pus to be found; however the systemic condition of my patient was not so good.

The temperature and pulse never gained the height of first examination, but he suffered from loss of appetite, mental worry and malaise. This, however, yielded to a tonic treatment of iron and tincture nux vomica, and recovery was uninterruped.

My diagnosis of psoas abscess is based, first, on the relief of pain in a few hours after operation, when sedatives had failed; second, complete subsidence of swelling after removal of grumous blood which had not become fetid; third, possibly traumatic in origin, being shocked three months previously; fourth, three or four days growing pain over right side before severe attack.

At no time was there any abnormal discharge from bowels, the only inconvenience being from the bladder, which was easily relieved by bi-tart. potass. in lemonade.

Since recovery he has gained his usual weight, resumed usual labor, and says he is as "stout as ever."

There is, at present, no trace of tenderness, deep-seated over the cecum or over the colon, at any point.

CHAS. A. JEFFERIES, A.B., M.D.

